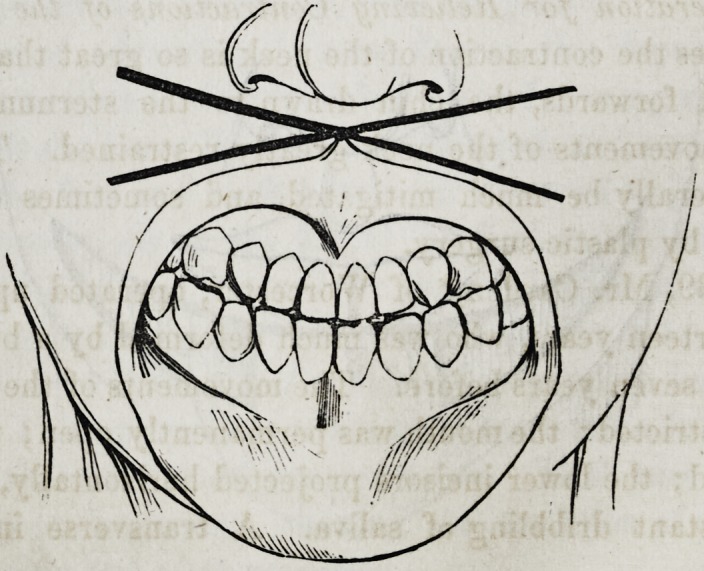# On Plastic Operations for the Restoration of the Lower Lip and for the Relief of Several Deformities of the Face and Neck

**Published:** 1857-07

**Authors:** Thomas P. Teale

**Affiliations:** Surgeon to the Leeds General Infirmary.


### ARTICLE XV.

*On Plastic Operations for the Restoration of the Lower Lip and for the Relief of several Deformities of the Face and Neck.* By Thomas P. Teale, Surgeon to the Leeds General Infirmary.

There is, perhaps, no department of surgery in which the practice of the present day contrasts more favorably with that of the age immediately preceding, than the plastic.

In the hope of advancing, in some degree, this department, I invite the attention of the profession to the following series of cases in which the lower lip has been restored, and several deformities of the face and neck have been removed or lessened.

In the Transactions of the Royal Medical and Chirurgical Society for 1855, I was honored by the publication of a paper on a plastic operation for the restoration of the lower lip, which was exemplified by three cases in which the operation had been performed. I now propose to relate these cases more fully, and some others that have since occurred to me. Each case will be illustrated by an engraving, showing the condition of the patient both before and after operation.

To prevent repetition in the relation of the cases, I shall first describe the several operations to which these patients have been subjected.

1. *Operation for the Restoration of the Lower Lip.*—The usual cause which renders this operation necessary is the contraction following deep and extensive burns of the neck. As contraction advances, the chin becomes drawn down to the sternum; the mucous membrane of the lower lip is turned outwards and drawn to the lower edge of the chin; the incisor teeth of the lower jaw gradually assume a horizontal direction, and are drawn much in advance of those of the upper jaw. In

AA. Lateral flaps formed of everted lower lip and cheek. B. Central portion of everted lower lip.

AA. Lateral flaps united in the median line, above the central portion of everted lower lip B. CC. Exposed surfaces left to granulate.

extreme cases the lower incisors take a direction almost horizontal. The tongue sometimes lolls out of the mouth, and the saliva is constantly dribbling away.

To relieve this sad condition the following operation is proposed:

Two vertical incisions, about three-quarters of an inch in extent, are made through the everted lip down to the bone. These incisions are so placed as to divide the upper portion of the everted lip into three parts—the middle being equal to one-half of the natural breadth of the lip, while the two lateral portions are each equal to one-fourth. From the lower end of each vertical incision the knife is carried in a curving direction outwards and upwards to a point situated about one inch from the angle of the mouth opposite to the second molar tooth of the upper jaw. The two flaps thus marked out and deeply incised are then separated from the bone, the mucous membrane uniting them to the alveoli being freely divided. Lastly, a bare surface is made along the alveolar border of the middle portion of the everted lip. The incisions being now completed, the lateral flaps are drawn upwards and united by twisted sutures to each other in the median line, and to the middle portion of the everted lip at their inferior border. In this way a new lip is, as it were, built upon the middle portion of the old one.

2. *Operation for Restoration of the Upper Lip.* The process of cicatrization sometimes reduces the upper lip to a narrow transverse band, drawn up close to the nose, leaving the upper teeth and gums exposed. This deformity interferes with the perfect closure of the mouth, and causes an unseemly aspect.

The contracted upper lip in the fifth case of the present series was restored to its natural size and function by the following operation :

A crucial incision is made (*en saltire,*) having its point of intersection immediately below the septum of the nose. Each limb of this incision is about one and a half inch in length. The two limbs on each side diverge moderately as they pass outwards to the cheek, and enclose between them an acutely angular flap of skin and other tissues. This crucial incision is extended deeply through the entire substance of the imperfect lip and the cheeks. The parts implicated in the incisions are then freely loosed from their attachments to the superior maxillary bone by the knife being passed upwards between the bone and the remnant of lip. The parts being thus detached, the two lateral angular flaps are drawn across the median line, dovetailing with each other, and thereby increasing the depth of the lip at the expense of its breadth. In this position the flaps are retained by one pin and twisted suture.

3. *Operation for Relieving Contractions of the Neck.*—In some cases the contraction of the neck is so great that the head is bowed forwards, the chin drawn to the sternum, and the lateral movements of the neck greatly restrained. These evils may generally be much mitigated, and sometimes completely relieved by plastic surgery.

In 1839, Mr. Carden,* of Worcester, operated upon a girl aged fourteen years, who was much deformed by a burn, which occurred seven years before. The movements of the head were much restricted; the mouth was permanently open; the tongue protruded; the lower incisors projected horizontally, and there was constant dribbling of saliva. A transverse incision was

* Transactions of Provincial Medical and Surgical Association, Vol. xii.

made throughout the entire extent of cicatrix in front of the neck. The chin was then drawn upwards, and every tense band connected with the cicatrix was divided until the head was relaxed nearly into its natural position. A flap of skin, three inches long and two and a half inches wide, was detached on each side from over the clavicle and chest. These were raised and united in front of the throat. The degree of improvement effected in this case, and tested by the lapse of four years, was highly gratifying.

Subsequently to the performance of Mr. Carden's operation, a similar proceeding was adopted in several cases, with great success, by Dr. Mütter,* of Philadelphia.

I have performed this operation in seven cases since August, 1848, and have witnessed it in some others by my colleagues at the Leeds Infirmary.

In all the cases which I have seen, there was a marked and most satisfactory improvement in the movements of the head and neck. The displacement of the lip was also in a greater or less degree mitigated by the operation on the neck, but in several of the cases this particular deformity remained to such an extent as to render a special operation for the restoration of the lower lip subsequently necessary.

In these autoplastic operations on the neck, it is of essential importance, as stated by Dr. Mütter, that the incision of the scar should extend from sound skin on one side of it to sound skin on the other, and that every band of adventitious fibrous tissue beneath the scar should be divided until the bottom of the wound discloses a loose healthy cellular tissue.

The flap to be transplanted may be taken from any neighboring portion of the neck, shoulder, or thorax, where healthy skin can be obtained. In one case, from lack of sufficient sound skin, I was under the necessity of including cicatrized skin in the flap.

The very accurate adaptation of the flap by suture, should be avoided, as great tension renders the flap liable to slough. It

* British and Foreign Medical Review, April, 1845.

is, therefore, better to be content with attaching the flap at its free extremity and one of its borders, and to leave the other border loose. Much may be done afterwards by careful dressing, during the healing process, to rectify any separation of the parts.

As far as I have observed, the transplanted flap rarely unites to the edges of the wound by the "first intention." All that is usually accomplished in the first instance is an organic union of the cellular surface of the flap to the parts beneath. The more close approximation of the edges of skin is obtained during the processes of granulation and healing.

When the bands of scar are so numerous or extensive as to require more flaps of skin than one to be inserted, it is better to repeat the operation at separate times. I saw much constitutional disturbance in one case, from the operation having been conducted on too large a scale in the first instance.

After the lapse of some months the transplanted portion of skin is generally found to have yielded to a process of stretching, so as to exceed considerably its original dimensions.

4. *Operation for Restoration of the Lower Eyelids.*—Eversion of the lower eyelid, its tarsal border being drawn far down the cheek, is a frequent result of contracted scars. Besides the revolting appearance caused by permanent ectropeon, the patient suffers habitually from a low form of inflammation of the conjunctiva and cornea, in consequence of these parts having been habitually deprived of the protection of the eyelid.

The eyelid in such cases may frequently be restored to its natural position by the following operation:

An incision is made across the cheek parallel to the displaced tarsal border, about three lines below it. The portion of skin between this incision and the edge of the tarsus is freely dissected upwards, along with the whole substance of the eyelid as far as the edge of the orbit. The eyelid thus loosened is placed in its natural position, and the chasm left thereby, is filled by a piece of skin transplanted from the side of the face. This operation succeeded perfectly in the right eye of William Bradby, the subject of the fifth case. It was attempted with only partial success in both eyes of John Leach, the subject of the fourth case. The want of complete success in this instance was owing to the total absence of any portion of sound skin in the neighborhood; on which account, I was obliged to transplant on each side, a piece of cicatrix, which having only low vitality sloughed to a considerable extent. In two other cases, not included in this series, the operation succeeded perfectly.

5. *Restoration of the Upper Eyelids.*—From the contraction of scars of the upper eyelids and forehead, the upper lids are sometimes everted, and their tarsal border is bound firmly to the superciliary ridge.

A plastic operation similar to that for the lower lids may be practiced with advantage in this deformity. In the case of John Leach, to be hereafter related, I operated on each of the upper lids, by making a transverse incision parallel to the tarsal border, at a distance of three lines above it. The substance of the eyelid was then dissected downwards and freely loosened from the edge of the orbit. The upper eyelid being thus restored to its natural position, the vacuity was filled by a piece of skin, transplanted from the temple. In both eyes the operation succeeded.—*Med. Times & Gazette.*

## Figures and Tables

**Figure f1:**
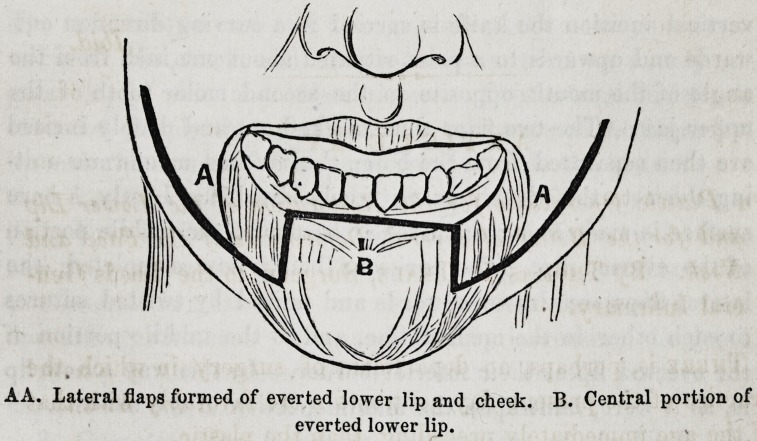


**Figure f2:**
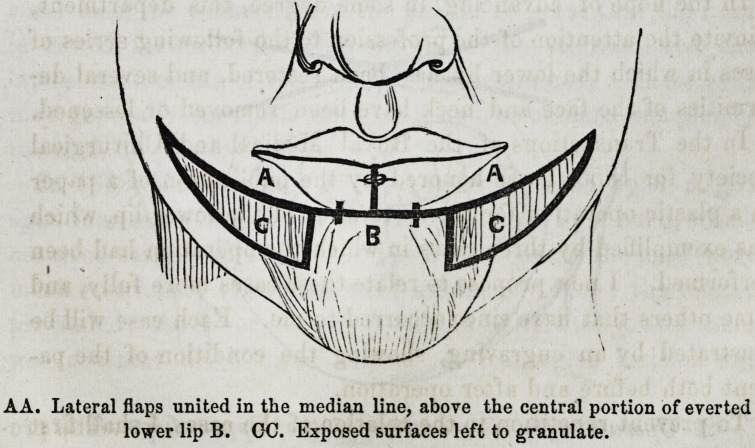


**Figure f3:**